# Central Sensitization-Based Classification for Temporomandibular Disorders: A Pathogenetic Hypothesis

**DOI:** 10.1155/2017/5957076

**Published:** 2017-08-28

**Authors:** Annalisa Monaco, Ruggero Cattaneo, Maria Chiara Marci, Davide Pietropaoli, Eleonora Ortu

**Affiliations:** MeSVA Department, University of L'Aquila, P.le S. Tommasi, 67100 L'Aquila, Italy

## Abstract

Dysregulation of Autonomic Nervous System (ANS) and central pain pathways in temporomandibular disorders (TMD) is a growing evidence. Authors include some forms of TMD among central sensitization syndromes (CSS), a group of pathologies characterized by central morphofunctional alterations. Central Sensitization Inventory (CSI) is useful for clinical diagnosis. Clinical examination and CSI cannot identify the central site(s) affected in these diseases. Ultralow frequency transcutaneous electrical nerve stimulation (ULFTENS) is extensively used in TMD and in dental clinical practice, because of its effects on descending pain modulation pathways. The Diagnostic Criteria for TMD (DC/TMD) are the most accurate tool for diagnosis and classification of TMD. However, it includes CSI to investigate central aspects of TMD. Preliminary data on sensory ULFTENS show it is a reliable tool for the study of central and autonomic pathways in TMD. An alternative classification based on the presence of Central Sensitization and on individual response to sensory ULFTENS is proposed. TMD may be classified into 4 groups: (a) TMD with Central Sensitization ULFTENS Responders; (b) TMD with Central Sensitization ULFTENS Nonresponders; (c) TMD without Central Sensitization ULFTENS Responders; (d) TMD without Central Sensitization ULFTENS Nonresponders. This pathogenic classification of TMD may help to differentiate therapy and aetiology.

## 1. Introduction

Chronic TMD is a frequent disorder in the general population. Its diagnosis is mainly clinical and the diagnostic criteria include signs of dysfunction of stomatognathic system and provoked and/or spontaneous pain of the joint and/or the stomatognathic muscles at rest or during function.

Pain, in particular, is necessary for the diagnosis of TMD. To date, evidence is inconclusive regarding instrumental biomarkers of TMD based on the anatomic and functional analysis of the stomatognathic system [1, 2]. For this reason, it has been suggested that the characteristics of pain in TMD could explain the pathogenesis of the disorder better than other mechanisms [3, 4]. For example, the concomitant presence of TMD and headache in many TMD patients suggests a clinical association between the diseases and a possible common pathogenesis of pain [5, 6].

The international classification of headache (ICHD) and that of TMD (RDC/DC) consider the characteristics of pain in headache and TMD and have introduced criteria to differentiate between them [7, 8]. However, the association between the two forms is greater than what random association could suggest and the clinical course of the one often follows that of the other [9, 10], suggesting a potential comorbidity that has also been hypothesized for TMD and other disorders characterized by chronic pain (e.g., fibromyalgia [FM], headache/migraine, irritable bowel syndrome [IBS], and low back pain [LBP]) [11]. Based on the hypothesis of a common central dysregulation in the modulatory pathways of pain, these disorders have been systematically classified as central sensitization syndromes (CSSs) [12], with TMD representing a specific CSS [13, 14].

*Central Sensitization Syndromes (CSSs).* CSSs are a group of disorders characterized by chronic nonneuropathic and nonnociceptive pain; the pain is not proportional to the type of injury/damage and it must be accompanied by the presence of neurophysiological/neuropathological phenomena (secondary hyperalgesia, allodynia). These disorders frequently lack histopathologic and/or instrumental evidence that can directly and proportionally explain the severity of pain and disability, and specific therapies are usually unsatisfactory in the short and long term [15, 16].

The nosologic category of CSSs is recent, and the list of disorders is in progress. Although originally used to justify the “chronic pain,” the current definition also embraces functional and cognitive impairment, such as those observed, among others, in IBS, insomnia, restless leg syndrome, multiple chemical sensitivities, and disorders characterized by affective and emotional symptoms (e.g., anxiety, panic, depression, and posttraumatic stress disorder) [17]. These syndromes share the pathogenic mechanism of CS. The CS is defined as “an amplification of neural signaling within the central nervous system (CNS) that elicits pain hypersensitivity” [15]:

Some neurophysiological characteristics of the neurons affected by CS are the generation or increase in spontaneous firing activity, the lowering of the activation threshold for their physiological stimuli, the more intense and longer activation after a nociceptive stimulus, and the development of larger receptive fields [18].

Due to the plastic properties of neurons, CS determines a change in the functional state of the CNS characterized by an increased release of excitatory transmitters and continuous activation of specific nervous pathways. Through functional changes that involve the amplification of the physiological transmission of impulses, any central neural structure can become the center of hyperexcitation and can trigger an altered response to afferences. Information exchanged among the CNS structures remains active after the termination of the peripheral phenomenon and can induce endocrine, motor, not physiologically oriented autonomic responses, and pain. Neurophysiological tests were conducted in the CSS to demonstrate the presence of secondary hyperalgesia and/or allodynia, which are considered markers of CS [16, 18–21].

From a biological point of view, the psychological phenomena related to chronic pain have a counterpart in the dysregulation of cognitive, neuromuscular, autonomic systems, and the endogenous opioid system [22].

Although common anatomic and functional patterns of CS among CSS are not yet clearly demonstrated [23], convincing data indicate that the Periaqueductal Gray (PAG) plays a key role in the maintenance of this state [24–30].

## 2. TMD and CSS

Chronic TMDs are characterized by chronic pain, which is also a characteristic of CSSs. The probability that pain becomes chronic is significantly related to the presence of spontaneous or provoked pain on palpation at more than one site of the body, both in TMD and in other CSSs [31]. In addition, TMDs are frequently associated with other CSSs [31–33]. For example, there is a significant correlation between TMD and Myofascial Pain Syndrome, Tension Type Headache/Migraine, FM, IBS, Chronic Fatigue Syndrome, Multiple Chemical Sensitivity, and posttraumatic stress disorder with childhood onset [34]. Moreover, TMD patients displayed higher CSI scores compared to other CSSs, such as FM and IBS [34, 35]. For this reason, authors suggested that TMD patients could suffer from generalized hyperexcitability in the CNS nociceptive pathways [31, 32].

TMD should be considered a multisystem disorder with the involvement and dysregulation of the sensory-motor, psychic, self, inflammatory, and immune systems [36], confirming the general pathogenetic hypothesis of a multisystemic genesis of chronic pain [37, 38].

TMD patients, in fact, showed consistent functional/structural changes in the thalamus and the primary somatosensory cortex. Additionally, functional and structural changes were frequently reported in the prefrontal cortex and the basal ganglia in TMD, suggesting the role of cognitive modulation and reward processing in chronic orofacial pain [39]. Some studies demonstrated that TMD patients suffer from a dysregulation in the autonomic nervous system (ANS) [40–42]. In addition, these subjects show a higher frequency of psychiatric disorders (anxiety, depression, alexithymia, and catastrophizing) [43–48].

Taken as a whole, these data suggest that TMD patients suffer of a dysfunction in the endogenous pain inhibition systems [49, 50] especially at the PAG level [51]. As with the CSSs, also for TMD it is difficult to find a specific physical and psychological marker that can give an account of all disorders, possibly because CS is not characteristic of a specific and unique nucleus or pathway, and it is possible that during the chronification process the involvement of different afferent and efferent systems entails the possibility of combining different outputs [38].

Sometimes the Central Sensitization starts from a peripheral injury and/or dysfunction; frequently, this is the case of chronic TMD in which muscle, dental occlusion, or temporomandibular joint (TMJ) dysfunction can be considered the peripheral triggers; the deep tissues (muscle, fascia, and joints) are the most powerful in determining the sensitization [52–54] producing enhancement of pain behavior and nociceptive neuronal activity through an alteration in the descending inhibitory or excitatory influences from structures such as the rostral ventromedial medulla (RVM) and a depressive effect on central opioid pathway [55]. This may explain why for a long time from the clinical point of view dental research has given special attention to the TMJ and/or muscles of stomatognathic system, believing these structures to be the origin of the TMD. In this sense, the idea was not completely wrong. Originally, the problem may have been triggered by acute or subacute pain of deep tissue of the stomatognathic system. The current literature, however, confirms that, once established, the CS becomes independent from injury or damage at the peripheral tissue level and maintains pain despite healing or disappearance of the original damage [18].

It is possible that the chronification process has an individual predisposition characterized by the inability to “extinguish” the memory circuits in the brain (nucleus accumbens/hippocampus/medial prefrontal cortex) triggered by the original injury [56].

In this case, the circuit would be continuously triggered by trivial, not necessarily painful, stimuli. Only for the fact that the circuit is active every stimulus would reinforce the memory process and the sensitization itself. Chronic pain is therefore to be understood as a “brain disease” and not as a peripheral disorder [57] although an improvement in peripheral conditions may contribute to the improvement of the central state [23].

This aspect would explain in part the current difficulties in the interpretation of specific TMD literature that deals with the search for an objective peripheral cause. Once the CS has been established for the effect of the original peripheral cause, the latter loses its function and is no more automatically related to the disorder.

From the clinical point of view, these observations could have an interesting implication on the diagnosis and, therefore, the therapy of TMD, configuring the possibility of a gradation of central involvement in TMD according to its chronicity and its comorbidity with other CSSs. Consequently, a modulation of intervention can be hypothesized that may range from a purely dental therapy (e.g., bite), in case of little or no CS, to drugs or psychological/psychiatric therapies when CS is prevalent.

Indirect evidence of the presence of CS in TMD subjects is given by the effectiveness of centrally acting drugs in a percentage of patients [23, 58]. The use of benzodiazepines [59, 60], tricyclic antidepressants [60, 61], beta blockers [62], gabapentinoids [63, 64], and melatonin [65, 66], in fact, has shown some effectiveness in reducing pain and other related symptoms (sleep disorders and/or affective-emotional disorders). Among others, melatonin is particularly studied as a therapeutic strategy in CSSs because of its many positive effects and the lack of side effects. It likely acts on reducing pain and allodynia through the activation of MT2 receptors at many sites of the CNS, particularly in PAG, a site of primary importance in the modulation of descending pain system [67, 68]. It has been recently shown that melatonin has analgesic effects in TMD, probably acting through the endogenous opioid and GABAergic systems [65, 66].

## 3. TENS (Transcutaneous Electric Nerve Stimulation)

TENS is widely used as a therapy for the control of acute and chronic pain [69–73].

It is likely that the uncertainties about its effectiveness are linked to different modality of study, administration, and application of the stimulus, especially in older works [74–77].

The mechanism of action is probably the activation of the endogenous opioids system, and particularly the brainstem PAG-RVM circuit.

Direct evidences by Sluka's group on animal have shown that TENS works on endogenous opioid and activates central inhibitory pathway [78–82]. This group has systematically clarified the mechanism of action of TENS using different modality of stimulation, among them the high frequency high amplitude (motor) and low frequency low amplitude (sensory) TENS, showing the central mechanism of these modalities of stimulation. Specifically, both modalities of stimulation improve secondary hyperalgesia and allodynia, considered marker of central effect. Only low frequency low amplitude TENS fails on primary analgesia, considered marker of peripheral effect [83]. On the other end, both high frequency high amplitude and low frequency low amplitude tens work at central level: they reduce the secondary hyperalgesia in rats if administered contralaterally to the site of chronic inflammation [84]. This data confirms that low frequency low amplitude TENS does not work on pain modulation at peripheral level. King and coll. stated that “different frequencies are only important with respect to reducing primary hyperalgesia but not secondary hyperalgesia. Increasing intensity above sensory threshold does not increase inhibition” [84, 85].

The location of the mechanism of action has been demonstrated by animal studies. Spinal administration of low dose of naloxone (at low dose naloxone works as specific antagonist of *µ* receptor of endogenous opioids) and naltrindole (antagonist of *δ* receptors) in arthritic rats prevents the antihyperalgesia after both low frequency low amplitude and high frequency high amplitude tens show that the *δ* and *µ* receptors are the target of stimulation. Particularly, at spinal level low frequency low amplitude TENS works on *µ* receptors while high frequency high amplitude TENS works on *δ* receptors [79]. The RVM *µ* receptor blockade with microinjection of naloxone reverses the low frequency low amplitude TENS effect on secondary hyperalgesia in rats. The RVM microinjection with naltrindole (*δ* receptor antagonist) did not affect secondary antihyperalgesia of low frequency low amplitude TENS, while it affected the antihyperalgesia due to high frequency high amplitude TENS [80].

Moreover, the RVM response to stimulation is under the control of specific areas of PAG: in animal model of chronic inflammation low frequency low amplitude TENS works on vlPAG (ventrolateral PAG) and does not work on dlPAG (dorsolateral PAG) [82]. Taken together, these animal studies confirm the central effect of TENS, specifically of low frequency low amplitude TENS, by PAG-RVM path of endogenous opioid.

The PAG-RVM circuit is part of the descending pain modulation system and is crucial in determining the coupling between the afferent and efferent responses towards excitement or inhibition [25, 86–92]. Authors suggested that probably the process of chronification is characterized by the shifting from the PAG-RVM on-cell to the PAG-RVM off-cell path [24, 93, 94]. In particular, PAG-RVM receives vagal and trigeminal afferents from the periphery, but also from supraspinal structures such as the hypothalamus, the amygdala, and the circuit that integrates the work of the lateral and medial prefrontal cortex [95].

The PAG-RVM is interleaved with the systems that are responsible for stress response, sensory integration, hormonal and motor somatosensory, and visceral response to pain [96–98] and is related to the centers controlling the arousal state, particularly the Locus Coeruleus (LC) [99–104]. In addition, the LC is involved in the maintenance of hyperalgesia and allodynia and participates in the expression of multiple pain modalities with descending facilitation from the RVM [105].

An alteration of this balance due to central or peripheral phenomena, such as an alteration in the occlusion [106–108], may induce a state of CS at this level, in part justifying the hyperarousal state in CSSs. LC has been also linked to a category of disorders characterized by imbalance of the arousal systems and alteration of tonic-phasic function of the LC, including disorders of affective-emotional sphere [109], chronic pain disorders [110, 111], and migraine [112, 113].

The data suggest that an imbalance between the arousal system and that of the descending pain modulation may be present in the CSSs. TENS may recognize its diagnostic and therapeutic rationale into the interaction with the circuits discussed above.

While the analgesic effect of TENS was thoroughly investigated, there are few works that dealt with its nonantalgic effects. A positive effect has been observed on sickness [114], on fatigue associated with FM [115], on distress [116], on upper [117] and lower limbs motility [116], on heart rate variability (HRV) [118], on pupil dynamics [50], on peripheral blood flow and cutaneous temperature [119], on memory and affective behavior [120, 121], and on EEG [122].

Taken together, although scattered, these data seem to suggest that effects on autonomic (cardiovascular, temperature, and skin conductance) and cognitive system are associated with analgesic effect of TENS, indicating widespread central, antalgic, and nonantalgic effects of this technique that could have utility in the management of CSSs.

## 4. Ultralow Frequency TENS (ULFTENS)

A particular type of TENS has been used for a long time in dentistry for a variety of purposes, including treatment of pain in TMD patients, prosthetic rehabilitation, and diagnosis and treatment in orthodontics [123–125]. It is defined as Ultralow Frequency TENS (ULFTENS) because of the frequency of stimulation (0.66 Hz), belonging to the field of ultralow frequencies (<20 Hz). In ULFTENS, electrical stimulation is applied bilaterally in the preauricular area to stimulate the fifth and seventh cranial nerves [126].

The analysis of the physical characteristics and neuromuscular effects of ULFTENS in dental practice is beyond the purpose of this article. Our hypothesis herein is the possibility that ULFTENS can be helpful in understanding the pathogenesis and helping differential diagnoses of TMD meant as a CSS. Classically, ULFTENS is delivered with amplitude that induces contraction of the elevator muscles, so that a little upwards movement of the jaw is obtained (so called low frequency and high amplitude TENS). The main purpose is to obtain reduction of pain and “relaxation” at rest of the stomatognathic muscles, especially the masseter, anterior digastric, and anterior and posterior temporalis muscles, in TMDs. The effect of stimulation is clinically assessed with the use of surface EMG of the cited muscles and with computed kinesiography of jaw movements to measure the amount of free space between the dental arches after ULFTENS, which allows for the comparison with the prestimulation condition [127–129]. A considerable amount of data suggests that pain improves after ULFTENS, the electrical activity at rest tends to decrease, and free space tends to increase [130, 131].

This assumption has been used to suggest that the ULFTENS acts through a double effect on muscle relaxation, favoring the peripheral metabolic exchange of contracted muscles, and the PAG-RVM circuit of pain, inducing the release of endorphins. On the other hand it is possible that not all stimulated subjects undergo a reduction in muscles contraction and an increase of free space [132]. In a lower proportion of subjects (5–15%), in fact, the EMG values increase and the free space decreases. This is in contrast with the idea that the ULFTENS obtains its effect through the “muscle relaxation” with a peripheral mechanism of action. More likely, the trigeminal stimulation causes a central effect that can highlight a generic predisposition to a “paradoxical” generalized response. To test the hypothesis that the responsible partly for ULFTENS effects on muscle relaxation and pain is the central sensory circuit, our research group has used ULFTENS with sensory amplitude to exclude muscle movement. Our data seem to suggest that sensory ULFTENS induces EMG reduction and increases free space in a probably centrally driven manner [133]. For better understanding of the phenomenon, we applied the same stimulation protocol checking the output of pupil dynamics instead of mandible muscle tonus and position. In this way, if the stimulation of sensory, not painful, component of V cranial nerve had an impact on the central systems controlling the arousal state, it would have been possible to get an effect on a system (pupil) not directly under the voluntarily neuromuscular system driven by V cranial nerve. Moreover, the pupil could be considered heterosegmental compared to the V pair of cranial nerve; its involvement by sensory ULFTENS may be considered a central effect of this modality of stimulation. Our data seem to confirm the hypothesis: sensory ULFTENS changes the pupil dynamics in the dark, in the light, and during the voluntary clenching of the teeth. Moreover, the response to sensory ULFTENS is different between healthy and TMD subjects. The latter seem to have difficulties in maintaining and recruiting the correct balance between the two branches of autonomous system controlling the pupil dynamics. Considering the relationship between the arousal system and the pupil dynamics we suggested that sensory ULFTENS works on arousal system but in different way in healthy and TMD subjects: particularly, in the TMD patients sensory ULFTENS seems not be able, compared to healthy subjects, to activate the inhibitory path coming from vlPAG or, alternatively, it seems to activate the dlPAG, instead of vlPAG, increasing the dysregulation between inhibitory and excitatory systems [41, 50]. This explanation agrees with other authors [24, 93, 94] about the different activation of RVM on off-cells driven by PAG activity. Next step of our study used the HRV as peripheral counterpart of central activity of inhibition system [134]. The goal was to test, in healthy subjects, the effect of sensory ULFTENS on the arousal system after acute mental stress. The hypothesis was that if sensory ULFTENS worked on the brainstem inhibitory component of the arousal system we would have seen, comparing subjects receiving and not receiving sensory ULFTENS, an effect on HRV without different perceived mental stress. Our results showed that subjects who received sensory ULFTENS have a lower activation of the system controlling the HRV than people who did not receive sensory ULFTENS by the same rate of subjective perceive mental stress. In other words, under mental stress the psychological component of the stress was comparable in the two groups, but the autonomic activation controlling the heart dynamics under mental stress was significantly lower in subjects receiving sensory ULFTENS. This data suggests that sensory ULFTENS worked on brainstem circuitry copying the output of heart dynamics independently from sovra brainstem circuitry analysing the mental stress [135].

Taken together these data agree with those works previously cited on low frequency low amplitude TENS suggesting its central effect, probably located at brainstem level. This location of the mechanism of action of sensory ULFTENS allows hypothesizing its use to evaluate the Central Sensitization at brainstem level.

Using this type of ULFTENS, it was possible to highlight the dysregulation of the ANS and of the pain modulation systems in TMD [50], suggesting that this technique can be used to evaluate the central component of the CSSs. In fact, sensory ULFTENS induces central modifications at both high and low frequencies [82]. Our data agree with those of Moran et al. [136], who stated that the sensory TENS has a significant effect in inducing hypoalgesia compared to placebo. The effects obtained in our work partly disagreed with the claims of Lauretti [137], who argues that high amplitude stimuli that cause intense muscle contraction are necessary to obtain a low frequency supraspinal effect with TENS. It is probable that the trigeminal territory stimulated with dental ULFTENS has different somatosensory central characteristics of signals integration than those found in other parts of the body, such as the dorsal lumbar and/or limbs, which can contribute to explain our results. For example, important direct connections have been demonstrated in rats between the nuclear trigeminal system and the PAG and, therefore, with the PAG-RVM system [87, 138, 139] and from these structures into areas of the ventrolateral orbital cortex, nucleus accumbens, or the amygdala: in the limbic or affective-motivational centers of the pain-related neural system [140].

**Figure 1:**
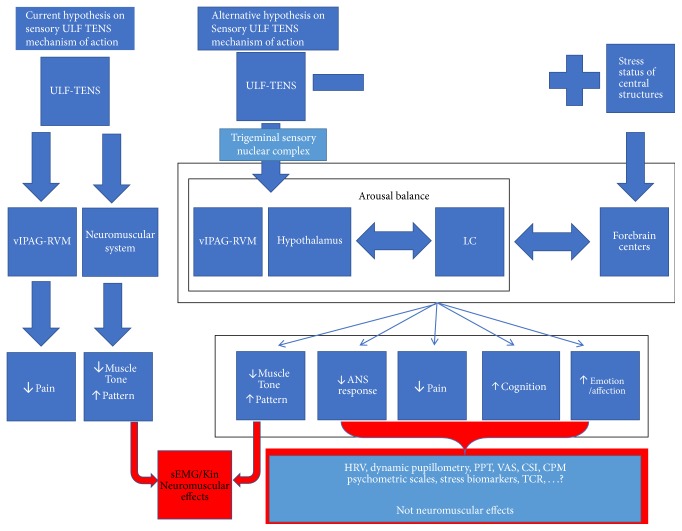
*Current and alternative mechanism of action of ULFTENS*. vlPAG-RVM: ventrolateral Periaqueductal Gray, LC: Locus Coeruleus, ANS: Autonomic Nervous System, sEMG/Kin: surface Electromyography and computed kinesiography of mandibular movements, HRV: Heart Rate Variability, PPT: Pressure Pain Threshold, CPM: Conditioned Pain Modulation, VAS: Visual Analogic Scale, and CSI: Central Sensitization Inventory.

The hypotheses on the mechanism of action of ULFTENS are summarized in Figure 1. The neuromuscular classical theory is linked to the hypothesis that electrical stimulation may reduce pain by acting on the central circuit (PAG-RVM) and simultaneously induce a relaxation of the neuromuscular system, secondary to the impulse-driven rhythmic movement and the reduction of catabolic substances via a pump effect, with improvement of tropism and, consequently, of the muscle tension. Our alternative hypothesis is that a system that controls the balance of arousal drives the individual's reaction. Among other structures, we hypothesize that this system is formed by the PAG, the periventricular nucleus of the hypothalamus and the LC. In turn, these subcortical structures would be under the control of superior brain centers, although able to create a feedback to stimulate or inhibit the cortical centers.

In the “normal” condition collaboration exists for the control of arousal between cortical and subcortical centers. Information transmitted through sensory ULFTENS reaches the nuclear trigeminal sensory complex and through the latter is projected to subcortical areas that control arousal (LC, hypothalamus, and PAG-RVM). Acute stress and pain lead to increased arousal (allostasis) followed by the temporary activation of peripheral responses mediated by the ANS as well as the inflammatory, immune, hormonal, and neuromuscular systems [141, 142].

It is likely that such action takes place by the “inhibition of the inhibition” of the “activation system” according to the hypothesis of Thayer [143, 144].

In these conditions, the ULFTENS would act through the balance of subcortical arousal circuit by enhancing the inhibition through the endorphin system and, thus, reducing the cortical activation induced by stress or pain. The action on peripheral targets will vary, since the paths that lead to the peripheral response are themselves varied and dependent on individuals. For this reason, it will be possible to obtain different combinations of peripheral effects to observe the reduction of muscle tone and the change of the neutral position of the jaw, the reduction of pain, the variation of the dynamics of the pupil, the increase of the heart rate variability, the reduction of the oxidizing molecules and antioxidant barrier in saliva and serum, or the changes in cognitive-emotional test in which.

In cases where the arousal system was not in suitable conditions to bear an additional stress (e.g., deficiency or dysregulation of the inhibitory systems observed under stress condition and chronic pain), the stimulation with ULFTENS may not have the above-mentioned “inhibitory” effect. Peripherals answers cannot be, therefore, those expected and they can show an opposite behavior, for example, an increase in muscle tone, a reduction of the free space, an increase of salivary and blood levels of oxidants and a decrease in antioxidant barrier, a reduction of heart rate variability, and a paradoxical response of the pupil.

By acting at the level of PAG-RVM component of the arousal control system, the ULFTENS would show the inability of this system to trigger the action of inhibition of endorphin circuitry if CS is present. The effects of sensory ULFTENS, both neuromuscular and not, are determined by the functional state of the general arousal system and by the subcortical system's ability to activate the appropriate stimulus-response coupling sequences.

In particular, this hypothesis is different from that traditionally suggested for the mechanism of action of ULFTENS, because it does not consider its effect on the muscles tone and on the dynamic pattern of jaw movements (free way space after sensory ULFTENS) as the response of the peripheral neuromuscular system or its inherent proprioceptive properties. Indeed, we hypothesize that all the ULFTENS effects are linked to the central anatomofunctional substrate of the arousal balancer, affected by the state of “normalcy” or “CS.” Consequently, sensory ULFTENS can highlight, through objective data (dynamic pupillometry, HRV, Pressure Pain Threshold, Conditioned Pain Modulation, e.g.,) and/or clinical techniques (VAS, Central Sensitization Inventory, Allodynia Symptoms Checklist, psychometric tests), not neuromuscular and those neuromuscular indirect effects studied by surface EMG/KIN that can contribute to accounting for the location of Central Sensitization.

## 5. Proposal for the Differential Diagnosis and Treatment of TMD

**Figure 2:**
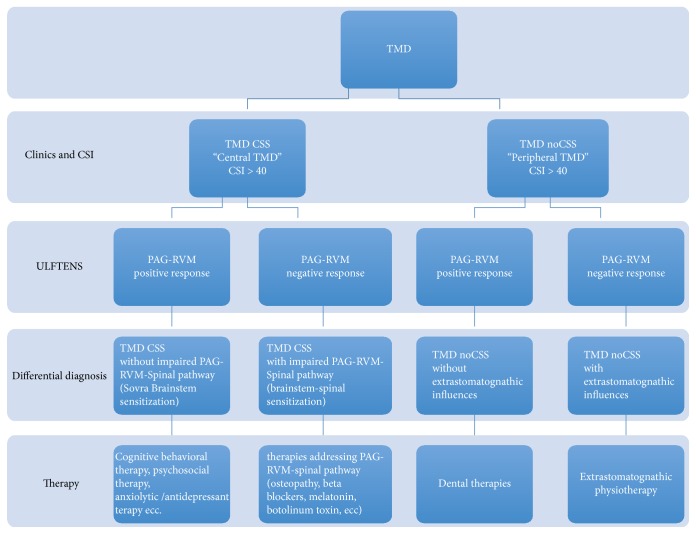
*Flow chart for differential diagnosis and therapy based on and response to ULFTENS*. CSI: Central Sensitization Inventory, TMD CSS: TMD with Central Sensitization, TMD noCSS: TMD without Central Sensitization, PAG-RVM: Periaqueductal Gray-Rostroventral Medulla.

The TMD therapy is based on a “ex juvantibus” principle. Typically, the therapy starts with hygienic recommendations, self-administered jaw exercises, and physiotherapy and progressively adds more specific drugs or dental interventions against the supposed cause of peripheral or central pain, up to the surgery on joint or orthodontics [143]. At present, irreversible treatments are not recommended, given the generally benign trend of the problem. The problem arises when the disorder becomes chronic and/or does not respond to conservative therapies. In this case the therapeutic choice is often based on a random choice and not on pathogenic hypothesis. In agreement with the above we would propose a working hypothesis, which should aim at overcoming the diagnostic and therapeutic impasse (Figure 2). It is possible that the concept of spectrum could be relevant to the TMD. The TMD spectrum would cover a range of disorders in which one extreme is characterized by acute forms with joint and muscle dysfunction and pain localized to one, strictly temporomandibular, district. Typically, these forms do not present difficulties from the clinical point of view for the differential diagnosis and/or therapy.

At the other end of the range there are chronic forms characterized by CS, no more necessarily linked to a peripheral trigger in the temporomandibular district, which frequently represent a diagnostic and therapeutic challenge. Between the two forms, there are probably mixed disorders in which, from time to time, the central or the peripheral component could prevail, but both are active and mutually influent. Although not yet fully elucidated, it is likely that CS has a key role in the mechanism that leads to chronicity. Furthermore, as previously argued, the pain in other locations than TMJ area or the presence of other forms of CSSs is induced to classify the TMD as a disorder characterized by CS.

The flowchart in Figure 2 aims to exemplify our hypotheses based on CS as an underlying pathogenetic factor, with sensory ULFTENS as a “provocation” test on the descending PAG-RVM system. PAG-RVM reacts to sensory ULFTENS as a function of its functional state of normality or CS. In case of normality, ventrolateral PAG and PAG-RVM circuit increase their activity and, among other effects, also induce muscle relaxation.

The first step after the clinical evaluation is the administration of the CSI, which has proved sufficiently sensitive and specific to discriminate the syndromes characterized by CS. The cut-off score of the questionnaire is 40. Subjects with a score above 40 have a high probability of belonging to the group of the CSSs. The CSI does not allow the specific localization of CS. In this sense, the CSI is generic. On the other hand, sensory ULFTENS identifies responders and nonresponders to stimulation. The clinical parameters that can be evaluated by sensory ULFTENS include the resting tone of the muscles and the amount of interocclusal free space. The first must be reduced and the second increased after ULFTENS: in this case, subjects are considered responders.

This criterion is probably insufficient because it only refers to a part of the activation of the PAG-RVM system and its quantification is not universally considered as reliable and specific for TMD. However, the information obtained indicates the response of the PAG-RVM circuit to the stimulation of the temporomandibular district.

Future work could improve this decision-making process by introducing additional, more appropriate tests to assess the autonomous response after sensory ULFTENS, for example, HRV, with or without cognitive/emotional tests.

Depending on the answer to the CSI and sensory ULFTENS, four diagnostic categories of TMD can be hypothesized.

(*1) TMD Subjects Characterized by Central Sensitization (TMD CSS) without Impairment of the PAG-RVM-Spinal Pathway.* These subjects are sensory ULFTENS responders, and their PAG-RVM-Spinal pathway responds to stimulation by activating the way of endogenous opioids, as expected from the hypothesis. According to this hypothesis, the CS is placed in areas overlying the brainstem, probably in cortical or subcortical structures responsible for the processing of pain and its cognitive and affective/emotional component. From a therapeutic point of view, it is likely that this group of people does not directly benefit of a dental approach. The most appropriate therapies should target the structures above the brainstem (cognitive-behavioral therapy, psychosocial therapy, therapy of anxiety and depression, etc.).

(*2) TMD CSS with Impairment of PAG-RVM-Spinal Pathway.* These subjects are sensory ULFTENS nonresponders; that is, the sensory ULFTENS does not evoke the expected response on behalf of the PAG-RVM-Spinal pathway that activates the endogenous opioid mechanism. Frequently, in sensory ULFTENS nonresponders a paradoxical response is triggered, with an increase in muscular electrical activity and a reduction or even absence of the free space. Sensory ULFTENS is not able to activate the inhibitory component of the PAG-RVM-Spinal pathway because of the prevailing excitatory response of the system. In this case, it is possible to suggest that CS is located at the level of the PAG-RVM-Spinal pathway. Therefore, this system should be the target of therapy. Appropriate therapies (high and low frequency TENS, osteopathic therapy, beta blockers, low intensity laser, melatonin, etc.) should be able to reverse the impairment of the system.

(*3) TMD without Central Sensitization (TMD noCSS) and without Extrastomatognathic Unbalance.* This is the classic case of acute TMD of dental interest, where the occlusal component, muscle, fascia, and joints of the stomatognathic system are cause of the problem, and there is no clinical evidence of CS. These subjects are sensory ULFTENS responders. From this basis, it can be argued that in chronic TMD an alteration in the trigeminal system afferents is present. It has been shown that the construction of reversible occlusion (bite) that maintains the spatial characteristics of the mandibular-cranial balance obtained under motor ULFTENS stimulation can significantly improve symptoms in a sample of acute TMD subjects [145]. At present, the pharmacological approach (nonsteroidal anti-inflammatory drugs [NSAIDs], benzodiazepines, and muscle relaxants) and physiotherapy of the stomatognathic system (manual therapy and physiotherapy exercises) are the first choice to control acute and subacute symptoms, which frequently arise during a period of fatigue or stress. If no results are obtained in this first phase of the treatment, a reversible dental treatment should start quickly to avoid the establishment of chronic pain and CS.

(*4) TMD noCSS with Extrastomatognathic Unbalance.* Also in this case, there is no evidence of CS, but the subjects are sensory ULFTENS nonresponders. It is possible that alterations in extra trigeminal districts in these subjects can influence the response to sensory ULFTENS of the fifth and seventh pairs of cranial nerves. In fact, afferents from extra trigeminal districts converge at the level of the caudal part of the spinal trigeminal nucleus, where they can contribute to the phenomenon of referred pain to the trigeminal area. Therefore, myofascial and articular disorders of extrastomatognathic districts may affect the trigeminal territory. Nonresponsiveness to sensory ULFTENS can depend on the anatomofunctional localization of the unbalance, namely, in districts not affected by sensory ULFTENS stimulation or, alternatively, because PAG-RVM system is working to reduce the inputs coming from extrastomatognathic system and could be no more engaged by sensory ULFTENS. In these subjects, it is possible to suggest a “postural” or physiotherapy approach aimed at rebalancing the extrastomatognathic structures responsible for symptoms referred to the trigeminal territory.

## 6. Warnings and Suggestions

The proposed model of sensory ULFTENS as a as PAG-RVM system provocation technique and as a diagnostic tool for different types of TMD is, at present, only hypothetical. Further studies are needed to shed light on this topic, particularly studies of neuroimaging or animal studies exploring the anatomofunctional sites that interplay in different types or stages of TMD.

Another limitation of the present study is inherent to the current idea of CSSs. In fact, a unique physiopathogenic pattern grouping all syndromes included in this classification into one entity has not yet been demonstrated. The term CS is too general, including any plastic or functional phenomenon that can involve any nervous structure, area, or CNS nucleus. It can also be attributed to multiple structures simultaneously.

Future works are also needed to associate specific tests with specific CSSs. In our case, we have hypothesized that sensory ULFTENS could act on one possible site of CS, the PAG-RVM system, which has a crucial role in the descending modulation of pain, hyperalgesia, and allodynia. In this our hypothesis is completely missing an essential step, which is to test the effect of sensory ULFTENS on allodynia and hyperalgesia in individuals belonging to the four diagnostic and therapeutic categories suggested.

TMD has to be considered a group of etiologically or, at least, pathogenetically different disorders. Without classifications that include these differences, the therapies are “casual” and not “causal.” In our work we do not want to support one or another therapy; we only hypothesized that the current therapies could be grouped in different way according to the proposed pathogenic-based classification. We proposed any “not already used” therapy. All the cited therapies have their “scientific” bibliography; all the TMD therapies, of course, are debated.

Future work should focus on this objective to assess whether individuals belonging to the four categories are different in terms of pain, hyperalgesia, and allodynia, and if sensory ULFTENS helps in differential diagnosis and, therefore, in the choice of the appropriate therapy.

## 7. Conclusion

Chronic TMD are a challenge for dentistry. The traditional clinical and research approach based on the injury and dysfunction of the stomatognathic system is no longer suitable to provide a convincing pathogenetic theory that could satisfactorily guide therapy. Thus, it seems useful to change perspective towards an interesting new possibility, represented by the study of chronic pain as a “central disorder,” originating from maladaptive learning and plasticity secondary to a peripheral dysfunction, the so called CS -, but quickly living its own independent life. Further clinical and basic research are needed to better understand the degree and type of involvement of anatomofunctional CNS sites in chronic TMD.
